# How German Hospital Ships Are Fitted

**Published:** 1917-02-10

**Authors:** 


### HOW GERMAN HOSPITAL SHIPS ARE FITTED.

[We have received the following article from a correspondent who has translated it from one by Dr. Weber, of the German Navy. We have already given some account of British ships fitted for hospital purposes, and think the following short account may prove interesting to many readers.]

Since efficiency on a man-of-war is hampered by the sick, it is easily seen that the aim of the fighting ship has always been to get rid of its sick and wounded, as these are a hindrance in action. This led at first to the brutal practice of throwing the dead overboard, as the fighting man was affected by the sight of the dead and dying, and later to the thought of bringing the sick and wounded under more favourable conditions than those to be found aboard a fighting ship. This idea was the beginning of the hospital ship. We can trace hospital ships back into the seventeenth century, although they served only for the removal of the sick and wounded after action, and had nothing else in common with the present hospital ship. Much later came the hospital ship with equipment designed for the care of the sick.

Hospital ships, on account of their different spheres of action, have to be divided into two classes—the auxiliary hospital ships, and the hospital ships proper. For auxiliary hospital ships small steamers have been utilised, which are easily managed and can be used not only in receiving sick from the ships, but also for either receipt or discharge of patients directly from or to docks. Suitable heating, ventilating, and lighting facilities are a foregone conclusion. The *personnel* and equipment of these ships are somewhat meagre—two medical officers—since the stay of patients is only short, limited to the time necessary for transfer to permanent quarters. Bed accommodation on these ships is provided for from 50 to 100 persons.

The hospital ships proper are vessels of from 5,000 to 6,000 tons displacement. They are good sea-boats, have suitable speed to follow a fleet, and are equipped with all the hygienic comforts of the up-to-date passenger ship. The different navies satisfy their need for hospital ships by the equipment, for that purpose, of merchant vessels. The German navy, in accordance with previously laid-down plans, converted merchant ships to the number of seven into auxiliary hospital ships, and six, with an average bed capacity of 300 patients, into hospital ships. These ships, converted into hospital ships in a short space of days, show in their interior already their changed mission. In accordance with the provisions of the second Hague Conference, they are painted white, with a one and a-half metre wide green stripe in the case of Government ships, and a red stripe in the case of ships fitted out by benevolent societies or private individuals. Besides the national flag, they also carry the Geneva flag.

The main feature of these ships are the wards, which generally occupy the two upper decks, providing not less than fifteen cubic metres of air space for each patient. The beds are either stationary, arranged with due allowance for the ship's motion, or swinging cots fastened to iron stanchions. Smaller wards for the accommodation of infectious diseases, mental and critical cases, and sick officers are also provided. Among other important features are the light and well-ventilated operating rooms, with anæsthetising rooms, dressing rooms, etc., usually located on the upper deck. An *x*-ray room is an absolute necessity. The dental care unavoidable in cases of injuries to the jaw necessitates a dental room with complete equipment for the treatment of such cases. The medical cases are usually in wards not adjacent to those for surgical, skin, eye, and ear cases, but are located next to the dispensary, and connect with a room for electrical and hydro-therapeutical treatments. It is hardly necessary to mention that each ward has its own bathroom facilities. The different lavatories are located next to the wards occupied by the infectious diseases, which latter also requires a disinfecting plant. Other necessary adjuncts of hospital ships are ample kitchen space, cold storage facilities, laundry, repair shop for instruments, sewing-room, and storage rooms for medical and surgical supplies. A library for the crew and for professional reference is also provided.

Of great importance on a hospital ship is an easily operated arrangement for the transfer of sick either from or to the ship, and for this purpose a double swinging cot is used, hung from a boom. On the ship itself, elevators take care of this problem. A life-saving apparatus is also included in the equipment. The *personnel* of the ship consists usually of the senior medical officer and three juniors, a pharmacist, and a dentist, also hospital orderlies and Red Cross nurses.

